# *AcJAZ2L2* confers resistance to kiwifruit bacterial canker *via* regulation of JA signaling and stomatal immunity

**DOI:** 10.1093/hr/uhaf215

**Published:** 2025-08-22

**Authors:** Zupeng Wang, Zhenting Sun, Hui Pan, Wenyi Li, Lili Huang, Faming Wang, Qiong Zhang, Xiaofen Yu, Dawei Li, Li Li, Caihong Zhong

**Affiliations:** State Key Laboratory of Plant Diversity and Specialty Crops, Wuhan Botanical Garden, Chinese Academy of Sciences, Wuhan 430074, China; Wuhan Botanical Garden, Chinese Academy Of Sciences, Wuhan 430074, China; State Key Laboratory of Plant Diversity and Specialty Crops, Wuhan Botanical Garden, Chinese Academy of Sciences, Wuhan 430074, China; Wuhan Botanical Garden, Chinese Academy Of Sciences, Wuhan 430074, China; State Key Laboratory of Plant Diversity and Specialty Crops, Wuhan Botanical Garden, Chinese Academy of Sciences, Wuhan 430074, China; Wuhan Botanical Garden, Chinese Academy Of Sciences, Wuhan 430074, China; State Key Laboratory of Plant Diversity and Specialty Crops, Wuhan Botanical Garden, Chinese Academy of Sciences, Wuhan 430074, China; Wuhan Botanical Garden, Chinese Academy Of Sciences, Wuhan 430074, China; Northwest A&F University, Yangling 712100, China; Guangxi Institute of Botany, Chinese Academy of Sciences, Guilin 541006, China; State Key Laboratory of Plant Diversity and Specialty Crops, Wuhan Botanical Garden, Chinese Academy of Sciences, Wuhan 430074, China; Wuhan Botanical Garden, Chinese Academy Of Sciences, Wuhan 430074, China; State Key Laboratory of Plant Diversity and Specialty Crops, Wuhan Botanical Garden, Chinese Academy of Sciences, Wuhan 430074, China; Wuhan Botanical Garden, Chinese Academy Of Sciences, Wuhan 430074, China; State Key Laboratory of Plant Diversity and Specialty Crops, Wuhan Botanical Garden, Chinese Academy of Sciences, Wuhan 430074, China; Wuhan Botanical Garden, Chinese Academy Of Sciences, Wuhan 430074, China; State Key Laboratory of Plant Diversity and Specialty Crops, Wuhan Botanical Garden, Chinese Academy of Sciences, Wuhan 430074, China; Wuhan Botanical Garden, Chinese Academy Of Sciences, Wuhan 430074, China; State Key Laboratory of Plant Diversity and Specialty Crops, Wuhan Botanical Garden, Chinese Academy of Sciences, Wuhan 430074, China; Wuhan Botanical Garden, Chinese Academy Of Sciences, Wuhan 430074, China

## Abstract

Kiwifruit bacterial canker, caused by *Pseudomonas syringae* pv. actinidiae, poses a critical threat to global kiwifruit production. Previous studies implicated jasmonic acid (JA) signaling in kiwifruit responses to this pathogen; however, the molecular mechanisms underlying JA-mediated regulation remain largely unclear. Here, we identified and characterized AcJAZ2L2, a pivotal jasmonate-signaling regulator that confers substantial resistance against *P. syringae* pv. actinidiae. Transcriptomic profiling coupled with consensus co-expression network analysis revealed that AcJAZ2L2 expression is uniquely up-regulated in resistant kiwifruit cultivars after pathogen infection. Functional validation through genome editing with the clustered regularly interspaced short palindromic repeat-associated protein 9 nuclease and, through transgenic overexpression, confirmed the essential role of AcJAZ2L2 in resistance. Specifically, lines overexpressing AcJAZ2L2 displayed markedly reduced disease symptoms, lower pathogen colonization, and decreased stomatal density, whereas knockout lines exhibited increased susceptibility. Mechanistically, AcJAZ2L2 directly interacts with AcMYC2-like transcription factors, repressing downstream JA-responsive genes (AcVSP2L1 and AcVSP2L2) and maintaining stomatal closure to prevent pathogen entry. Promoter analysis further revealed cultivar-specific allelic divergence that drives differential AcJAZ2L2 transcriptional activation, explaining genotype-dependent resistance levels. Our findings establish a novel JAZ–MYC regulatory module that links JA signaling to stomatal immunity in kiwifruit and provide precise genetic targets for breeding cultivars with enhanced resistance to bacterial canker.

## Introduction

Kiwifruit has become a major specialty fruit crop worldwide [1]. It is well known for its high vitamin C content and balanced nutritional composition, including minerals, dietary fiber, and health-promoting metabolites [2]. Currently, it has seen a substantial increase in cultivation area to 432 825 hectares with an annual production exceeding 6 million tons (http://faostat.fao.org). However, the onset of the pandemic and the proliferation of kiwifruit bacterial canker disease (KBC) caused by *Pseudomonas syringae* pv. *actinidiae* (Psa) have severely impacted the entire kiwifruit industry and global trade [3–6]. The pathogen Psa is a member of the *P. syringae* complex, which encompasses numerous significant plant pathogens responsible for various diseases in both wild and cultivated plants [7]. Psa can be divided into five biovars (biovar 1–5) based on their physiological and biochemical attributes, geographical origins, and genomic characteristics [4, 5, 8]. Biovar 3, which exhibits the highest virulence toward kiwifruit, is identified as the primary cause of the global pandemic affecting kiwifruit [4, 9, 10]. However, no effective method has been found to prevent and control the pandemic of kiwifruit bacterial canker disease [9, 10]. Consequently, it is imperative to elucidate the resistance mechanisms against KBC and to cultivate new germplasms exhibiting enhanced resistance [8, 11].

Plant hormones, including jasmonic acid (JA), salicylic acid (SA), and abscisic acid (ABA), play a pivotal role in modulating plant immunity [12, 13]. Significant variations were observed in the levels of JA, SA, and ABA among various resilient kiwifruit cultivars/species following Psa inoculation. This suggests a potential role for these hormones in determining the resistance or tolerance of kiwifruit to Psa [14, 15]. Following Psa infection, both the susceptible *Actinidia chinensis* cultivar ‘Hongyang’ (HY) and the highly resistant *A. chinensis* var. *deliciosa* cultivar ‘Jinkui’ (JK) demonstrated an increase in ABA and SA accumulation [14]. Conversely, the concentration of JA rose in HY but declined in JK, suggesting that the JA signaling pathway significantly influences the resistance/tolerance of kiwifruit to Psa [14]. Additionally, another study confirmed that there were significant differences in JA concentrations across various kiwifruit species following Psa [15]. Upon infection with Psa, the accumulation of JA was observed in the susceptible species *A. chinensis* while a decrease was noted in the resistant species *Actinidia arguta* [15]. The treatment of methyl jasmonate (MeJA) led to a significant increase in the density of the Psa endophytic population within kiwifruit leaves, exhibiting a 7.4-fold surge compared to nontreated Psa-inoculated plants, suggesting that JA suppresses the resistance of kiwifruit to Psa [16].

Previous studies have demonstrated that the JASMONATE-ZIM DOMAIN (JAZ) family proteins play a crucial role in modulating plant immune responses to pathogen invasions by modulating the JA- or SA-mediated signaling pathways [17–22]. These JAZs act as inhibitors of the JA signaling pathway by interacting with the MYC transcription factor (TF) and regulating downstream JA-responsive genes, notably the Vegetative Storage Protein (VSP) family, such as VSP1 and VSP2 in *Arabidopsis* [17–22]. VSP genes are recognized as typical markers of JA pathway activation and are generally induced by JA-responsive TFs such as MYC2 [23–25]. Concurrently, they play a crucial role in plant immune responses against invasions by *P. syringae*, anthocyanin accumulation, stomatal development, ripening, and senescence in plants [18, 26, 27]. In *Arabidopsis*, all 12 JAZ proteins contain the ZIM and jas domains, which enable them to interact with various TFs to regulate a range of biological processes [28]. In contrast, JAZ proteins can serve as the direct target of effector proteins produced by plant pathogens, significantly reducing plant resistance against these pathogens [18, 19, 27]. The HopZ1a effector secreted by *P. syringae* triggers the degradation of JAZ proteins through their acetylation [19], while the HopX1 effector facilitates JAZ protein degradation via its cysteine protease activity [29]. *AtJAZ2* can enhance *Arabidopsis* resistance to *Pseudomonas* invasion by inhibiting the stomatal re-opening process [18]. The tomato ortholog of *AtJAZ2* also prevents stomatal re-opening and enhances tomato resistance to *P. syringae* pv. *tomato* (Pto) DC3000 [17]. These studies confirm that *AtJAZ2* and its orthologs in different plant species have conserved functions in regulating plant resistance to *P. syringae*.

Previous studies have demonstrated that the expression patterns of several JAZ genes in kiwifruit are significantly altered upon Psa, implying a potential role of these JAZ genes in modulating kiwifruit resistance to Psa infection [30, 31]. However, the functional role of JAZ genes in kiwifruit responses to Psa has yet to be experimentally verified. In this study, we initially identified three homologs of *AtJAZ2* in kiwifruit. Transcriptome analysis revealed that among the three genes, only the *AcJAZ2L2* gene exhibited differential expression in kiwifruit materials with different resistance levels. Functional investigation of this gene revealed its significant enhancement of kiwifruit resistance to Psa infection and its impact on the dynamic alterations of kiwifruit leaf stomata. Our results suggested that *AcJAZ2L2* was one of the key regulatory genes controlling kiwifruit resistance to Psa, thereby providing a basis for future breeding efforts aimed at enhancing kiwifruit resistance to Psa.

## Results

### MeJA-treatment increased kiwifruit susceptibility to Psa infection

**Figure 1:**
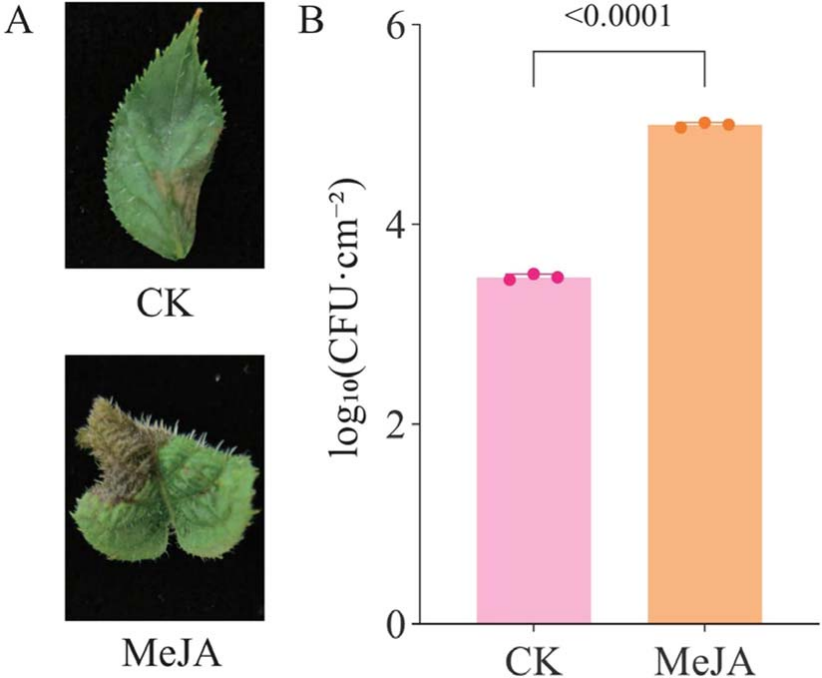
JA treatment reduces kiwifruit resistance to Psa. (A) Representative symptoms of kiwifruit treated with JA or a control solution (CK) following Psa incubation. (B) Bacterial growth was quantified at 14 days postinoculation (dpi) by serial dilution and plate count quantification. Bacterial counts were determined by dilution plating and colony forming units (CFU) per centimeter squared of leaf area. Differences between two groups were evaluated with a two-tailed Student’s *t* test.

Previous studies have suggested that JA levels are elevated in hypersensitive kiwifruit cultivars but reduced in tolerant/resistant cultivars upon *P. syringae* pv. *actinidiae* (Psa) infection, indicating a potential detrimental effect of JA on kiwifruit resistance to Psa [14, 15]. To evaluate the role of JA in kiwifruit resistance to Psa infection, we treated leaves of the susceptible cultivar HY with 100 μM MeJA, followed by inoculation with Psa. The treatment with MeJA led to a noticeable increase in lesion size (Fig. 1A), and there was also a significant rise in the number of Psa colonies per unit area in the MeJA-treated samples in comparison to the control samples (Fig. 1B). These results provided evidence that exogenous MeJA treatment enhanced kiwifruit susceptibility to Psa infection.

### Expression characteristics of *AcJAZ2L2* in kiwifruit in response to Psa

**Figure 2:**
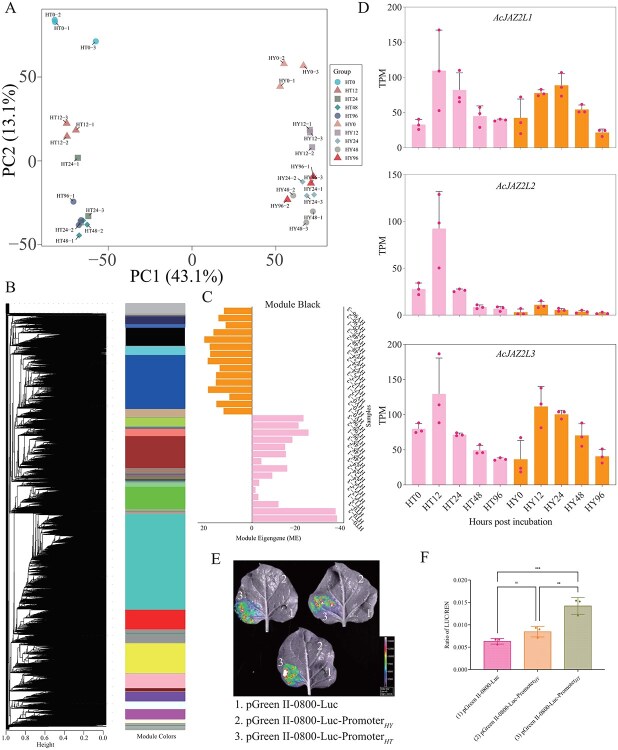
Identification of *AcJAZ2L2* as a key JA-responsive regulator in kiwifruit resistance to Psa. (A) PCA of transcriptomes from resistant (HT, *A. eriantha*) and susceptible (HY, *A. chinensis*) cultivars across five infection time points (0–96 hours postinoculation, HPI). Points represent replicates, colored by group. (B) CCNA of 33 045 genes reveals 21 stable modules. Dendrogram shows hierarchical clustering of genes; color bars denote module assignments. (C) Module eigengene expression for Module Black displaying divergent temporal dynamics between HT and HY. (D) Expression patterns of three *AcJAZ2*-*like* genes (*AcJAZ2L1/L2/L3*) across time points. (E) Transient expression assays in *N. benthamiana* leaves co-transformed with the promoter–reporter constructs and a constitutive 35S:Renilla (REN) control. Representative images show LUC and REN activities visualized separately. (F) Quantification of LUC/REN ratios for each construct in HY and HT cultivars of kiwifruit. Data are means ± SD (*n* = 3 biological replicates). ns = not significant. ^**^P < 0.01, ^***^P < 0.001 by Student’s *t* test.

We next sought to identify JA-related candidate genes potentially mediating JA’s role in kiwifruit responses to Psa. To this end, we re-analyzed the transcriptomes of a resistant cultivar (HT, originated from *Actinidia eriantha*) and a susceptible cultivar (HY, originated from *A. chinensis*) at five postinoculation time points (0, 12, 24, 48, and 96 hours) [32]. First, the sequencing reads were mapped to the Red5 reference genome, and the mapping rates were calculated (Table S2). The results indicated that the HT material had a mapping rate of approximately 80%, while the HY material had a rate of around 95% (Table S2). This discrepancy may be attributable to the fact that these two materials were derived from distinct kiwifruit species (Table S2). Nevertheless, both mapping rates were sufficient for subsequent transcriptome [32] analyses. Expression analysis identified 10 872 differentially expressed genes (DEGs) (Table S3). From these, the top 100 DEGs, which ranked by adjusted *P* value, were selected for heatmap visualization, and the results revealed distinct genotype-specific expression patterns between HT and HY (Fig. S1). Principal component analysis (PCA) of these DEGs demonstrated a clear separation of samples based on both cultivar and infection time, underscoring the divergent transcriptional responses in HT and HY (Fig. 2A).

To further identify gene clusters associated with kiwifruit canker resistance and key candidate genes related to the JA signaling pathway, we conducted a consensus co-expression network analysis (CCNA) [33]. The TPM expression matrix comprising 33 045 genes was used as the input for CCNA. Subsequently, 1000 random subsamples (each containing 80% of the genes) were generated to serve as input files for sub-WGCNA analyses (Table S4), and random parameters were employed for WGCNA (Table S5). After 1000 iterations, a weighted adjacency matrix was constructed and used as input for WGCNA to obtain the consensus clusters (Fig. 2B). In total, 21 modules were identified and correlation coefficients among genes within each module were significantly higher than those among genes across different modules, indicating the stability and consistency of these identified modules (Fig. 2B and Fig. S2). Eigengenes for each module indicated that Module Black displayed pronounced differences in temporal expression between HT and HY, reflecting its core involvement in genotype-specific transcriptional reprogramming during Psa infection (Fig. 2C, Fig. S3 and Table S6). We annotated the functions of 1506 genes in this module and identified a candidate gene, *Acc09801*, associated with the JA signaling pathway (Fig. S3 and Table S7). This gene encodes a typical JAZ protein (Table S7). Subsequent phylogenetic analysis involved a comprehensive search for JAZ family members in the kiwifruit genome and comparison with the full JAZ repertoire of *Arabidopsis thaliana* (Fig. S4). Three kiwifruit *TIFY* proteins, including *Acc09801*, clustered with SlJAZ2, AtJAZ1, and AtJAZ2 (Fig. S4), all of which contain the conserved TIFY and CCT_2 domains (Fig. S4). These three genes were designated *AcJAZ2-like 1* (*AcJAZL1*, *Acc12940.1*), *AcJAZ2-like 2* (*AcJAZL2*, *Acc09801.1*), and *AcJAZ2-like 3* (*AcJAZL3*, *Acc23402.1*) (Fig. S4). To assess the evolutionary conservation of AcJAZ2L2 across the genus *Actinidia*, we retrieved its orthologs from 15 high-quality genomes covering nine kiwifruit species. Multiple-sequence alignment revealed that the proteins are highly conserved, sharing 91.6%–100% amino acid identity (mean = 95.88%; Fig. S5A and B). Both signature domains—the TIFY motif (residues 35–39) and the Jas motif (residues 189–209)—were completely invariant in every genome examined (Fig. S5A). A maximum-likelihood phylogeny based on the full-length sequences recapitulated the established *Actinidia* species relationships (Fig. S5C). Finally, Ka/Ks analysis yielded ratios ranging from 0.095 to 0.336 (average = 0.269), all well below 1, indicating strong purifying selection and underscoring the functional conservation of AcJAZ2L2 throughout diverse kiwifruit germplasm (Fig. S5D).

We subsequently evaluated the expression profiles of the three *AcJAZ2*-*like* genes and found that only *AcJAZ2L2* was differentially regulated between HT and HY at early infection stages (12 and 24 HPI), which were consistent with the CCNA results, suggesting that *AcJAZ2L2* may serve a critical role in kiwifruit resistance to Psa (Fig. 2D). To explore the basis of this differential regulation, we cloned an approximately 1.3-kb promoter fragment from each cultivar and assessed promoter activity using a dual-luciferase reporter assay in *Nicotiana benthamiana* (Fig. 2E and Fig. S6). Strikingly, the HT promoter drove significantly higher luciferase expression than the HY promoter (Fig. 2E and F). These findings indicated that cultivar-specific promoter activity underlay the divergent transcriptional regulation of *AcJAZ2L2*, supporting its pivotal function as a negative regulator in kiwifruit canker resistance *via* the JA pathway.

### *AcJAZ2L2* positively regulated kiwifruit resistance to bacterial canker disease

**Figure 3:**
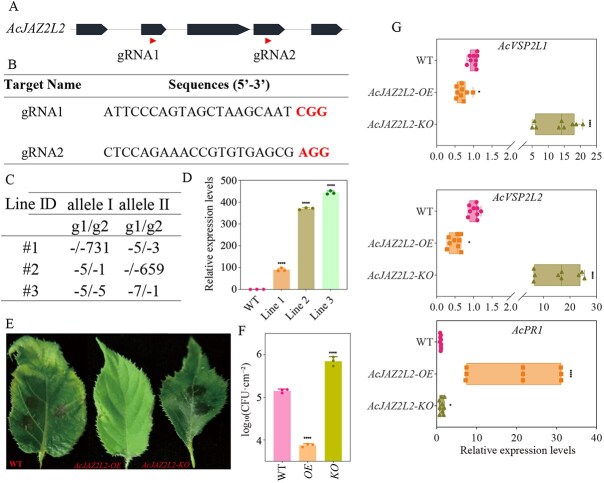
*AcJAZ2L2* positively regulated kiwifruit resistance to bacterial canker disease. (A) Schematic diagram of the *AcJAZ2L2* gene model showing the target sites of gRNA1 and gRNA2. (B) Sequences of the two guide RNAs (gRNA1 and gRNA2) used to target the *AcJAZ2L2* gene. (C) Detection of CRISPR/Cas9-induced mutations at the *AcJAZ2L2* locus by Sanger sequencing of PCR amplicons spanning the target sites in transgenic lines. (D) Relative expression levels of AcJAZ2L2 in overexpression (OE) lines determined by RT-qPCR. Data are means ± SD (*n* = 3 biological replicates). (E) Representative symptoms of WT, KO, and OE kiwifruit lines treated with Psa. (F) Quantification of bacterial population levels (log 10 CFU/cm^2^) on WT, KO, and OE lines. Data are means ± SD (*n* = 3 biological replicates). Different letters indicate significant differences (*P* < 0.05). (G) Relative expressions of *AcVSP2L1/L2* and *AcPR1* in *AcJAZ2L2* OE and KO lines with Psa infection. Differences were evaluated with two-tailed Student’s *t* tests, and multigroup comparisons with one-way ANOVA followed by Tukey’s HSD test (normality and homogeneity of variance verified in GraphPad Prism; significance thresholds ^*^P < 0.05 and ^***^P < 0.01).

To verify the role of *AcJAZ2L2* gene in modulating kiwifruit resistance to Psa, we employed CRISPR-Cas9 technology to induce sequence-specific mutations within *AcJAZ2L2* gene (Fig. 3A and B). Two guide RNAs (gRNAs) targeting *AcJAZ2L2* were designed and cloned into a singular gene editing vector to construct a paired gRNA construct following established protocols, which was then introduced into kiwifruit through *Agrobacterium*-mediated transformation as previously outlined [34]. Specific primers of gene editing vectors (SP-DL/SP-R) were utilized for amplification to detect positive lines, with polymerase chain reaction (PCR) products subsequently validated via Sanger sequencing (Table S1 and Fig. S7). Three independent positive lines carrying bi-allelic edits in the *AcJAZ2L2* gene were validated and subjected to subsequent characterization (Fig. 3C and Fig. S8).

Furthermore, we generated *AcJAZ2L2* overexpression lines to validate the function of *AcJAZ2L2*. The full coding sequence of *AcJAZ2L2* was amplified and cloned into the pOE-3xFLAG vector, followed by transformation into kiwifruit as previously described. T-DNA integration was confirmed by specific primers flanking the insertion site (FLAG-F/FLAG-R) (Fig. S8), and three independent transgenic lines were selected. We further analyzed the expression levels of *AcJAZ2L2* in transgenic lines, which were significantly higher than those in wild type (Table S1, Fig. 3D and Fig. S9).

Incubation experiments were performed on Psa to evaluate the resistance of gene editing and overexpression lines of the *AcJAZ2L2* gene, as well as to observe the phenotypes of various materials at 14 days postincubation (Fig. 3E). The leaves of both the wild-type and gene-edited lines exhibited necrotic lesions. However, these typical phenotypes were absent in the overexpression lines of *AcJAZ2L2* (Fig. 3E). Furthermore, we quantified the Psa endophytic population density across the lines. The Psa densities in the overexpression lines of *AcJAZ2L2* were significantly reduced compared to those in wild type (*P* < 0.001) (Fig. 3F). Conversely, the Psa endophytic population density in gene editing lines was significantly elevated in comparison to the wild type (Fig. 3F). These findings indicated that overexpression of *AcJAZ2L2* enhanced kiwifruit resistance to Psa infection. We performed relative fluorescence quantifications of JA-related genes and resistance-related genes in different genetic materials of *AcJAZ2L2*. The results showed that the expression levels of the JA response genes *AcVSP2L1/L2* were inhibited by *AcJAZ2L2*, while *AcPR1* was significantly induced (Fig. 3G). These findings suggest that *AcJAZ2L2* may enhance resistance to kiwifruit canker by inhibiting the expression of JA-related genes.

### *AcJAZ2L2* influenced kiwifruit stomatal development and stomatal dynamics

**Figure 4:**
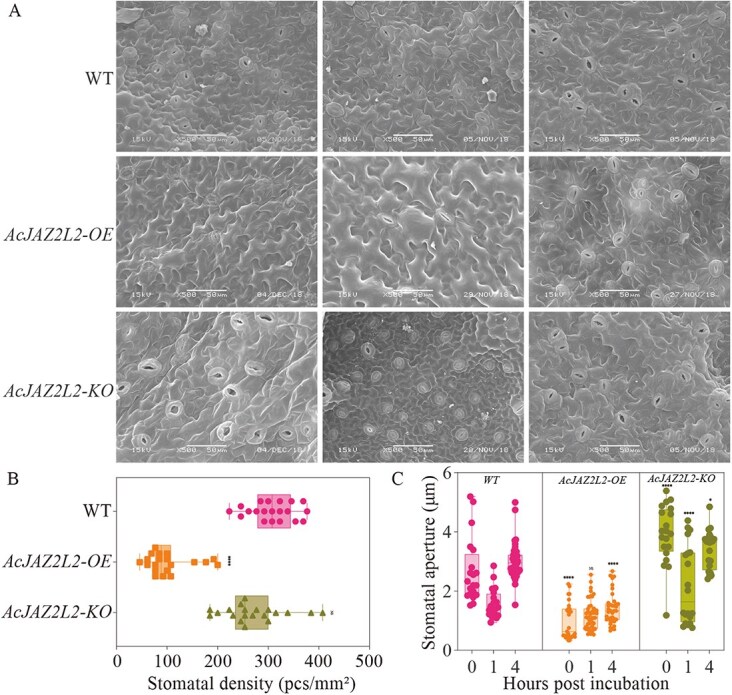
Stomatal dynamics in kiwifruit treated with Psa. (A) Representative micrographs showing stomatal density on kiwifruit leaf surfaces of wild type (WT), *AcJAZ2L2* overexpression (AcJAZ2L2-OE), and *AcJAZ2L2* knockout (AcJAZ2L2-KO) kiwifruit lines. Scale bars = 50 μm. (B) Quantification of stomatal density (number of stomata per millimeter squared) on the abaxial leaf surface of WT, OE, and KO lines. Different letters indicate significant differences (*P* < 0.05). (C) Stomatal aperture sizes (μm) in WT, OE, and KO lines measured at 0, 1, and 4 hours postincubation (HPI). Differences were evaluated with two-tailed Student’s *t* tests, and multigroup comparisons with one-way ANOVA followed by Tukey’s HSD test (normality and homogeneity of variance verified in GraphPad Prism; significance thresholds ^*^P < 0.05 and ^***^P < 0.01).

Previous studies have confirmed the role of *AtJAZ2* and *SlJAZ2* in regulating stomatal dynamics to prevent pathogen invasion during *P. syringae* invasion [17, 18]. To determine whether *AcJAZ2L2* could regulate kiwifruit stomatal dynamics in response to Psa infection, we measured the stomatal density and dynamics of kiwifruit leaves at three time points (0, 1, and 4 hours) after Psa inoculation (Fig. 4A). We quantified the stomatal density in the gene editing and overexpression lines (Fig. 4B). Stomatal density was significantly reduced in overexpression lines relative to the wild type (*P* < 0.0001) (Fig. 4B). However, no significant difference was observed between the gene editing lines and the wild type (Fig. 4B). We then examined stomatal dynamics in kiwifruit leaves during Psa infection three times (Fig. 4C). At 1 hour post-Psa inoculation, we noted that the stomatal apertures of both the wild-type and all transgenic lines were constricted (Fig. 4C). The stomatal apertures of the wild type showed a significant increase at 4 hours post-Psa incubation, indicating that Psa triggered stomatal reopening (Fig. 4C). Conversely, the stomatal apertures in the overexpression lines remained consistent at 4 hours postinoculation compared to those at 1-hour postinoculation. This suggests that Psa did not induce stomatal reopening in these lines (Fig. 4C). The sustained stomatal closure in the overexpression lines could potentially inhibit Psa internalization due to the reduced endophytic populations of Psa in these lines (Figs 3F and 4C).

### AcJAZ2L2 suppressed JA-responsive gene expression via direct interaction with AcMYC2-like proteins

**Figure 5:**
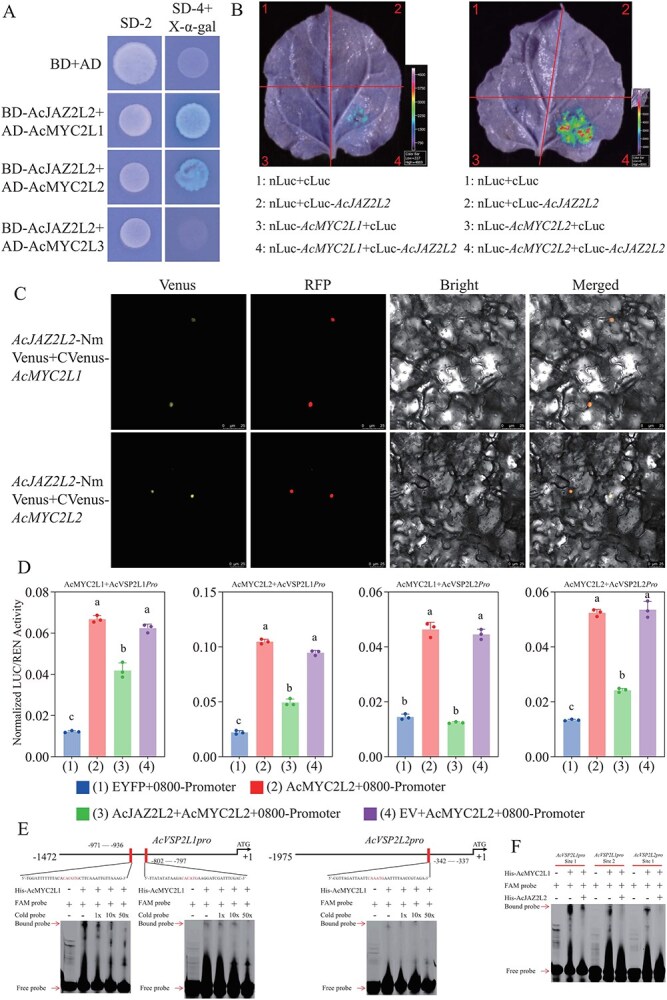
AcJAZ2L2 directly interacted with AcMYC2 TFs in kiwifruit. (A) Yeast two-hybrid assays showing interactions between AcJAZ2L2 and AcMYC2L1/L2/L3 proteins. Yeast co-transformants were plated on SD/−Trp/−Leu (DDO) medium to select for combined bait and prey vectors, and interactions were detected by growth on SD/−Trp/−Leu/-His/−Ade (QDO) medium and by x-α-galactosidase (x-α-gal) activity. (B) Firefly luciferase complementation imaging assay in *N. benthamiana* leaves. Luminescence intensity indicates interaction between nLuc-AcMYC2L1/L2 and cLuc-AcJAZ2L2. 1: Negative control; 2: AcJAZ2L2 self-interaction; 3: positive control (AcMYC2L1/L2); 4: AcMYC2L1/L2 interaction with AcJAZ2L2. (C) BiFC assays in kiwifruit leaves. Fluorescent signals indicate interactions between AcJAZ2L2-NmVenus and cVenus-AcMYC2L1/L2 in the nucleus. RFP was co-transformed as a nuclear marker, and merged images indicate co-localization.(D) *AcJAZ2L2* suppressed AcMYC2-like-mediated activation of JA-responsive promoters in kiwifruit. Dual-luciferase reporter assays in *N. benthamiana* leaves co-infiltrated with: (1) Empty vector (EV) + *AcVSP2L1* or *AcVSP2L2* promoter-LUC (negative control), (2) *AcMYC2L1* or *AcMYC2L2* + *AcVSP2L1* or *AcVSP2L2* promoter-LUC, (3) *AcMYC2L1* or *AcMYC2L2* + AcJAZ2L2 + *AcVSP2L1* or *AcVSP2L2* promoter-LUC, (4) EV + *AcMYC2L1* or *AcMYC2L2* + *AcVSP2L1* or *AcVSP2L2* promoter-LUC. Firefly luciferase (LUC) activity was normalized to Renilla luciferase (REN) to account for transfection efficiency. Data represented mean ± SD (*n* = 3 biological replicates). Different lowercase letters indicate statistically significant differences (one-way ANOVA, Tukey’s HSD test, *P* < 0.05). (E) AcMYC2L1 binds to specific motifs in the AcVSP2L1 promoter, and AcJAZ2L2 disrupts this interaction. EMSA using His-tagged AcMYC2L1 protein and FAM-labeled probes corresponding to MYC2-binding motifs in the *AcVSP2L1* and *AcVSP2L2* promoters. Competition assays with unlabeled probes (1×, 10×, 50× molar excess) confirmed binding specificity. (F) AcJAZ2L2 inhibited AcMYC2L1 binding to the promoters of *AcVSP2L1* and *AcVSP2L2*. Co-incubation of AcJAZ2L2 with AcMYC2L1 significantly reduced DNA–protein complex formation.

JASMONATE-ZIM DOMAIN (JAZ) proteins function as co-repressors by inhibiting the transcriptional activities of downstream regulators. Among these regulators, the basic helix–loop–helix (bHLH) TFs MYC2/3/4 have been identified as central mediators of JAZ functions in the JA signaling pathway [35]. Phylogenetic analysis of their full-length protein sequences, together with *Arabidopsis* bHLH family members, revealed three kiwifruit bHLH genes that cluster closely with *Arabidopsis* MYC2/3/4/5, which we designated AcMYC2L1, AcMYC2L2, and AcMYC2L3 (Fig. S10). To test whether AcJAZ2L2 interacts with these MYC2-like proteins, we performed yeast two-hybrid (Y2H), bimolecular fluorescence complementation (BiFC), and luminescence complementation assays (LCA). The Y2H results showed that AcJAZ2L2 could bind AcMYC2L1 and AcMYC2L2, but not AcMYC2L3 (Fig. 5A). This interaction was further confirmed by LCA in *N. benthamiana* leaves, which demonstrated that AcMYC2L1 and AcMYC2L2 physically associate with AcJAZ2L2 *in planta* (Fig. 5B). A subsequent BiFC assay in kiwifruit tissue corroborated these findings (Fig. 5C).

**Figure 6:**
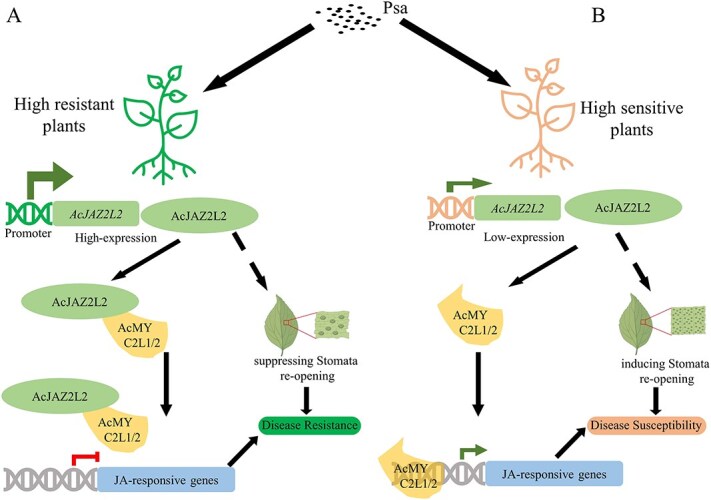
Proposed model for the role of *AcJAZ2L2* in determining resistance to Psa in kiwifruit. (A) In high-resistance plants (HT), strong *AcJAZ2L2* promoter activity drove elevated transcript levels, enabling *AcJAZ2L2* to sequester AcMYC2L1/2 TFs. This suppresses downstream JA-responsive genes and inhibits stomatal reopening, thereby blocking Psa entry. (B) In susceptible plants (HY), reduced *AcJAZ2L2* promoter activity led to insufficient AcJAZ2L2 protein. Without this suppression, AcMYC2L1/2 activated JA-responsive genes, promoting stomatal reopening and facilitating Psa.

We next examined how AcJAZ2L2 and AcMYC2-like proteins modulate JA-responsive genes in kiwifruit by focusing on *AcVSP2L1/L2*. Promoters of *AcVSP2L1* and *AcVSP2L2* (1472 and 1950 bp, respectively) were cloned for functional testing (Table S1). These promoters were introduced into a dual-luciferase reporter system, and co-expression assays revealed that *AcMYC2L1* and *AcMYC2L2* enhanced *AcVSP2L1* and *AcVSP2L2* promoter activities, whereas overexpression of *AcJAZ2L2* significantly suppressed this induction (Fig. 5D and Fig. S11). These observations indicated that *AcJAZ2L2* repressed *AcVSP2L1* and *AcVSP2L2* by interacting with *AcMYC2L1/L2*.

To determine whether *AcMYC2L1/L2* directly target the *AcVSP2L1* and *AcVSP2L2* promoters, we used PlantCARE to predict *MYC2* recognition motifs, identifying two sites within the *AcVSP2L1* promoter and one site in the *AcVSP2L2* promoter (Fig. 5E). Electrophoretic mobility shift assays (EMSAs) using purified recombinant *AcMYC2L1/L2* proteins confirmed specific binding to these promoter fragments (Fig. 5E). Addition of *AcJAZ2L2* protein reduced the DNA–protein complex, suggesting that AcJAZ2L2 competes with AcMYC2L1/L2 for binding (Fig. 5F). Collectively, these data demonstrated that *AcJAZ2L2* suppresses JA-responsive gene expression by directly interacting with AcMYC2L1/L2, thereby restraining their transcriptional activation of *AcVSP2L1* and *AcVSP2L2*.

## Discussion

Kiwifruit bacterial canker disease remains one of the most challenging factors limiting the sustainable development of the kiwifruit industry worldwide [36, 37]. Clarifying the underlying molecular mechanisms of resistance is fundamental to developing effective strategies for breeding canker-resistant kiwifruit cultivars [38]. JA signaling pathways are increasingly recognized as critical modulators of plant immunity, particularly in response to bacterial pathogens. Nevertheless, detailed molecular components through which JA signaling specifically mediates kiwifruit resistance to bacterial canker remain largely unknown [15]. JAZ proteins, as well-characterized repressors in the JA pathway, have been implicated broadly in plant–pathogen interactions, yet their precise roles in kiwifruit–Psa interactions require further clarification [39]. Nevertheless, the molecular mechanisms through which kiwifruit JAZ genes influence resistance genes remain ambiguous.

The effects of MeJA treatment on plant resistance to different types of pathogens (biotrophic, hemibiotrophic, and necrotrophic microorganisms) vary; our results showed that MeJA treatment significantly reduced kiwifruit resistance to kiwifruit bacterial canker disease (Fig. 1). *AtJAZ2,* along with its homologous genes, serves as a crucial co-repressor within the plant’s JA signaling pathway [17]. It collaborates with an array of other genes, including MYC2/3/4, to regulate the JA signaling pathway effectively [17, 18, 29, 35, 40–44]. Comparative transcriptome analysis of resistant and susceptible kiwifruit genotypes following Psa infection revealed that AcJAZ2L2, a putative regulator within the jasmonate signaling network, exhibits contrasting expression dynamics (Fig. 2). This divergence suggested that AcJAZ2L2 might modulate host defense by fine-tuning JA-responsive genes.

Our functional analyses strongly supported that AcJAZ2L2 positively regulated canker resistance, highlighting its potential utility in targeted breeding efforts (Fig. 3). The functional conservation of AcJAZ2L2 across diverse plant species such as *Arabidopsis* and tomato implies its central evolutionary role in JA-mediated defense responses [17, 42]. In *Arabidopsis*, *AtJAZ2* typically exerts its repressive effect on the JA signaling pathway by interacting with AtMYC2/3/4, thereby inhibiting the expression of downstream JA-responsive genes activated by AtMYC2/3/4 [35, 45]. Similarly, in kiwifruit, we found that *AcJAZ2L2* could interact with two kiwifruit *AcMYC2*-like genes, affecting the expression of downstream JA-responsive genes such as *AcVSP2L1/L2* (Figs 3G and 5). Consequently, we inferred that *AcJAZ2L2* also regulated the expression of JA-responsive genes through interactions with *AcMYC2*-like genes.

The remarkable conservation of AcJAZ2L2 protein sequences observed across diverse *Actinidia* species suggested evolutionary constraints, reflecting its essential functional role in immune modulation (Fig. S5A and B) [40]. These findings indicated that the core biochemical function of AcJAZ2L2 has been maintained throughout *Actinidia* evolution, suggesting that cultivar-specific disease outcomes were unlikely to stem from coding changes. Instead, differential promoter architecture likely underpinned the cultivar-specific transcriptional regulation, offering critical targets for future molecular breeding or genome-editing strategies. This high degree of protein sequence conservation, coupled with our functional data, underscored *AcJAZ2L2* as a promising, broadly deployable target for marker-assisted selection and genome-editing strategies aimed at enhancing bacterial-canker resistance across diverse kiwifruit germplasm.

Importantly, our findings emphasized that AcJAZ2L2 integrated hormonal signaling with stomatal immunity, reinforcing physical barriers to pathogen entry [17, 18, 35]. Stomata, as natural openings, are the main entry points for Psa [46], and a lower stomatal density can thus reduce the possibility of Psa. Overexpression of *AcJAZ2L2* appeared to reinforce stomatal immunity in kiwifruit by preventing pathogen-triggered stomatal reopening and thereby limiting bacterial entry (Fig. 4). Intriguingly, transgenic lines also exhibit reduced stomatal density, suggesting that AcJAZ2L2 may intersect with developmental programs governing guard cell differentiation (Fig. 4). Altogether, these data supported a model in which *AcJAZ2L2* served a dual role—modulating both the developmental patterning of stomata and their dynamic aperture responses during pathogen attack. Elucidating the downstream targets of AcJAZ2L2 in guard cell signaling and development will be crucial for unraveling its multifaceted contribution to kiwifruit defense.

Promoter sequence variation between resistant and susceptible cultivars appeared to drive distinct *AcJAZ2L2* expression patterns, revealing *cis*-regulatory elements as promising breeding targets (Fig. 2E and F). These results suggested that upon Psa infection, the differential promoter activities result in elevated expression of *AcJAZ2L2* in HT but not in HY. In HT, the elevated levels of *AcJAZ2L2* can inhibit the expression of JA-responsive genes by interacting with *AcMYC2*-like TFs, thereby enhancing resistance to Psa (Fig. 6A). In contrast, the low expression levels of *AcJAZ2L2* in HY fail to repress JA-responsive genes, resulting in susceptibility to the pathogen (Fig. 6B).

## Conclusion

In conclusion, our findings explained the contrasting responses of ‘Huate’ (HT) and HY to Psa infection at the transcriptional level. Differential promoter activity of *AcJAZ2L2* was a critical determinant of resistance, likely through regulation of downstream JA-responsive genes via the AcJAZ2L2-AcMYC2L1/L2 module. These results underscored the importance of transcriptional regulation in plant immunity and suggest that modifying the promoter activity of key regulators offers a promising strategy for enhancing disease resistance in crops.

## Materials and methods

### Plant materials and treatments

The seeds of *A. chinensis* var. *chinensis* HY and *N. benthamiana* used in this study were obtained from the Wuhan Botanical Garden, Chinese Academy of Sciences. All tobacco seedlings, kiwifruit seedlings, and transgenic materials were grown in a greenhouse at 23°C–25°C under long-day conditions (16 hours light, 8 hours dark). Samples collected from individual plants were considered biological replicates.

### Bacterial strains and growth conditions

Psa3 strains C48 were cultivated in Lysogeny Broth (LB) at 20°C under continuous shaking at 180 rpm [30]. *Escherichia coli* strain TOP10 was used for gene cloning and vector construction. TOP10 cells were cultured in LB supplemented with appropriate antibiotics at 37°C. The concentrations of antibiotics incorporated into selective media were as follows: kanamycin at 50 μg/ml. *Agrobacterium* strains (EHA105) were streaked onto LB plates, which were supplemented with rifampin and other essential antibiotics. These plates were then incubated at a temperature of 28°C for 2 days after being retrieved from a −80°C freezer. To commence the liquid culture, the bacteria were introduced into a 5-ml YEP liquid medium, which was also supplemented with the appropriate antibiotics. This mixture was then incubated overnight at 28°C, with continuous shaking to facilitate growth. For long-term storage, the *Agrobacterium* strains were preserved at −80°C in a solution containing 25% glycerol.

### Infection assays

Three leaves from each plant were selected for processing, ensuring consistent leaf positioning for treatment, and marking the target leaves. A sterile pipette tip was employed to apply a bacterial suspension to both the upper and lower surfaces of the leaves, ensuring comprehensive coverage with 50 μl of bacterial suspension per leaf. Each mutant type underwent three replicates. The control group received an equivalent volume of fresh liquid KB medium. Post-treatment, tissue-cultured seedlings were placed back into the incubation chamber (with a 12-hour light cycle, daytime temperature at 25°C, nighttime temperature at 20°C, and humidity consistently above 70%). Leaf phenotypes were documented using a digital camera at 0 days (noninoculated), 2 days postinoculation, and 14 days postinoculation. Pathogen colonization was evaluated based on the methodology previously reported [47].

### Transcriptome analysis and gene expression

Raw reads (PRJNA514180) were acquired from the NCBI repository for the purpose of scrutinizing the expression profiles of *JAZs* in kiwifruit. The raw data underwent re-analysis, employing *A. chinensis* cultivar ‘Red5’ as the reference genome [48]. Raw reads were aligned utilizing HISAT2 v2.0.1 [49]; thereafter, transcripts were assembled and quantified employing STRINGTIE v2.1.5 [50]. The CCNA was conducted according to the previous study [33].

Sample collection, RNA extraction, and RT-qPCR were performed as previously described [51]. Briefly, leaves were collected at various time points following Psa inoculation, and total RNA extraction was conducted utilizing the Plant RNA kit (R6827, OMEGA). RT-qPCR experiments were performed according to the guidelines provided with the SYBR Premix ExTaq master mix (RR420A, Takara Bio), employing the *AcActin* gene as the reference gene, and the primers were synthesized by the Beijing Genomics Institute (Table S1).

### Plasmid construction

For the overexpression of *AcJAZ2L2* gene, the pOE-FLAG-DN vector was constructed. The coding sequence of JAZ2L2 was amplified from kiwifruit cDNA using gene-specific primers (Table S1). The PCR products were then cloned into the pOE-FLAG-DN vector using Gibson assembly, generating pOE-JAZ2L2-FLAG construct. The integrity of the constructs was confirmed by Sanger sequencing.

For the generation of gene editing vectors targeting *AcJAZ2L2*, a CRISPR/Cas9-based approach was employed. Two guide RNA (gRNA) sequences targeting specific sites within the *AcJAZ2L2* gene were designed using CRISPR design tools (http://crispor.tefor.net/). The gene editing vector for AcJAZ2L2 was constructed as described previously [52].

### Kiwifruit transformation

Kiwifruit transformation was performed following established protocols [52]. Transgenic kiwifruit plants were carefully acclimatized to soil conditions in a controlled growth chamber or greenhouse environment. Transgene integration and expression were confirmed by molecular analyses such as PCR and quantitative real-time PCR (RT-qPCR) (Table S1). Additionally, the functionality of the CRISPR/Cas9 system in inducing targeted mutations was assessed by sequencing genomic DNA from transgenic plants to detect desired editing events at the target loci (Table S1).

### Transient expression in kiwifruit and tobacco

Recombinant plasmids containing the target gene were transformed into *Agrobacterium tumefaciens* EHA105 or GV3101 competent cells (Weidi Biotechnology, Shanghai, China) according to the instructions. The cells were then spread on LB solid medium supplemented with 50 mg/ml kanamycin (Kan) and 25 mg/ml rifampicin (Rif) and incubated at 28°C for 2 to 3 days. Positive clones of *Agrobacterium* containing the desired plasmids were picked and cultured in LB liquid medium supplemented with corresponding antibiotics for 1 to 2 days to obtain *Agrobacterium* seed solution. The seed solution was then mixed with fresh liquid medium at a ratio of 1:100 for further expansion culture. The cultured *Agrobacterium* liquid was centrifuged to remove the supernatant, and the bacterial cells were collected by resuspending and washing with resuspension solution (10 mM MgCl_2_ + 10 mM MES-K + 100 μM acetosyringone), followed by centrifugation to remove the supernatant. The washed bacterial cells were collected and resuspended in resuspension solution to an OD600 of 0.8, then incubated at room temperature in the dark for 2 to 3 hours before use. Transient transformation of kiwifruit and *N. benthamiana* was performed by injecting 1 ml of *Agrobacterium* suspension taken up in a syringe without a needle into the back of the leaves. The kiwifruit and tobacco plants injected with *Agrobacterium* suspension were kept in the dark for 24 hours before being cultured separately at 23°C for 3 days.

### Yeast two-hybrid assay

The full-length coding sequences of AcJAZ2L2, AcMYC2L1, and AcMYC2L2 were amplified from kiwifruit cDNA using gene-specific primers and cloned into the pGADT7 (prey) and pGBKT7 (bait) vectors, respectively. The bait and prey constructs were co-transformed into the yeast strain Y2HGold using the lithium acetate method. Co-transformants were selected on synthetic dropout (SD) medium lacking leucine and tryptophan (SD/−Leu/−Trp) to ensure the presence of both plasmids. Interaction specificity was assessed on selective media lacking histidine, tryptophan, leucine, and adenine (SD/-His/−Trp/−Leu/−Ade/X-α-Gal).

### BiFC assay

BiFC assays were conducted using pDEST–VYNE/VYCE vectors [34]. The coding sequences of AcJAZ2L2, AcMYC2L1, and AcMYC2L2 were cloned into pENTR/D-TOPO entry vectors and recombined with pDEST–VYNE/VYCE vectors. *Agrobacterium* cultures containing BiFC constructs were infiltrated into kiwifruit leaves. After 48 hours, leaf samples were analyzed by confocal microscopy (Leica TCS-SP8). Positive interactions were observed as reconstituted Venus YFP fluorescence localized in the nucleus, indicating physical association between the bait and prey proteins.

### Bimolecular luminescence complementation assay

The bimolecular luminescence complementation (BiLC) assay was performed to investigate the protein–protein interactions between AcJAZ2L2 and its putative interactors, AcMYC2L1 and AcMYC2L2, as described by Xu *et al.* [53], with adaptations for our experimental design. Full-length coding sequences of AcJAZ2L2, AcMYC2L1, and AcMYC2L2 were amplified from kiwifruit cDNA and cloned into the pCAMBIA-nLUC and pCAMBIA-cLUC vectors, respectively. These vectors enabled the fusion of the proteins of interest with the N-terminal and C-terminal halves of firefly luciferase (nLUC and cLUC), respectively. *Agrobacterium tumefaciens* cultures containing the respective BiLC constructs were prepared and co-infiltrated into tobacco (*N. benthamiana*) leaves. Infiltrated leaves were maintained under controlled growth conditions for 48 hours to allow for protein expression and interaction. Following incubation, leaf samples were treated with luciferin substrate and luminescence signals indicating protein–protein interactions were detected using a luminometer. Luminescence intensity was quantified as a measure of the strength of interaction between AcJAZ2L2 and AcMYC2L1 or AcMYC2L2.

### Dual-luciferase assay

The putative promoter region of the AcJAZ2L2 gene was cloned from kiwifruit cultivars HY (1.2 kb) and HT (1.3 kb) genomic DNA into the pGreenII 0800-LUC vector. *Agrobacterium* cultures containing the pGreenII 0800-LUC constructs were infiltrated into tobacco leaves. After 48 hours, leaf samples were collected and firefly luciferase (LUC) and Renilla luciferase (REN) activities were measured using the Dual-Luciferase Reporter Assay System (TransGen, Beijing, China). Relative promoter activity in HY and HT cultivars was determined by comparing LUC/REN ratios. The assay was conducted in triplicate, and statistical analysis was performed to assess differences in promoter activity between cultivars.

### Electrophoretic mobility shift assays

Recombinant His-tagged AcMYC2L1, AcMYC2L2, and AcJAZ2L2 proteins were expressed in *E. coli* and purified by Ni-NTA chromatography. Biotin-labeled probes corresponding to MYC2-binding motifs in the AcVSP2L1 and AcVSP2L2 promoters were synthesized (Table S1). EMSAs were performed using the EMSA/Gel-Shift Kit (Beyotime). Protein–DNA complexes were resolved on native polyacrylamide gels and visualized by chemiluminescence.

### Stomata aperture measurement in kiwifruit

Stomatal density and aperture of kiwifruit leaves were observed using a scanning electron microscope (JSM-6360LV). Leaf samples were fixed in 2.5% glutaraldehyde for over 2 hours, followed by vacuum infiltration for two to three cycles. After fixation, samples were stored at 4°C for at least 12 hours. Samples were washed with 0.1 M phosphate buffer (pH 7.2) three times, with buffer replacement every 2 hours. Dehydration was performed using ethanol gradients (30%, 50%, 70%, 80%, 90%) for 20 minutes each, followed by five rounds of dehydration in anhydrous ethanol for 10 minutes each. Samples were transferred to sample holders for freeze drying. Gold coating and ion sputtering were applied to the samples before observation under SEM. Stomatal density, length, and width were observed and photographed in multiple random fields. SEM images were analyzed using SmileView software. Stomatal density was calculated by dividing the number of stomata in each field by the corresponding area. Stomatal length and width were measured in each field using the same software.

### Genome-wide identification of JAZ genes in kiwifruit

Kiwifruit genome data (*A. chinensis* and *A. eriantha*) were obtained from the Kiwifruit Genome Database (http://kiwifruitgenome.org). To identify putative JAZ gene family members, the protein sequences of previously characterized *Arabidopsis* JAZ proteins (AtJAZ1-AtJAZ13) were used as queries in BLASTp searches against the predicted kiwifruit proteome [54]. All nonredundant hits with *E* values <1e−5 and query coverage >50% were retrieved and confirmed to contain characteristic TIFY (TIFF/CGI-1 and LIsH) and Jas (JASMONATE ZIM-DOMAIN PROTEIN) motifs using the SMART (http://smart.embl-heidelberg.de/) and Pfam (http://pfam.xfam.org/) databases [55, 56]. For each predicted kiwifruit JAZ protein, phylogenetic analysis was performed to verify orthologs to *Arabidopsis* JAZs. Full-length protein sequences were aligned with MUSCLE, and the alignment was used to construct a maximum likelihood phylogenetic tree with 1000 bootstrap replicates in MEGA X [57, 58]. *Arabidopsis* JAZ proteins were included to assess clade membership of kiwifruit JAZ homologs. Gene models were further manually curated using RNA-seq data to confirm splice junctions and untranslated regions. Protein sets for 15 high-quality kiwifruit genomes were downloaded and screened for AcJAZ2L2 orthologs by BLASTP using the HY sequence (identity ≥70%, coverage ≥80%) [59]. The best single hit per genome was retained. All orthologs were aligned with MUSCLE; amino acid identity was calculated with EMBOSS needle, and the conserved TIFY and Jas motifs were verified with Pfam HMM searches. A maximum-likelihood phylogeny was reconstructed in MEGA X (1000 bootstraps), while Ka/Ks ratios were estimated from codon-aligned CDSs using KaKs_Calculator.

### Statistical analysis

Unless stated otherwise, data are presented as mean ± SD from three independent biological replicates. Differences between two groups were evaluated with a two-tailed Student’s test. Multiple-group comparisons were performed by one-way ANOVA followed by Tukey’s HSD test. Normality and homogeneity of variance were verified with Shapiro–Wilk and Levene tests, respectively, in GraphPad Prism 10. The significance thresholds are *P* < 0.05 and *P* < 0.01.

## Supplementary Material

Web_Material_uhaf215

## Data Availability

The data that support the results of this study can be found in this paper and its supplementary materials.
