# Supplementary material for: AcJAZ2L2 confers resistance to kiwifruit bacterial canker via regulation of JA signaling and stomatal immunity
Source: Hortic Res. 2025 Aug 22;12(11):uhaf215. doi: 10.1093/hr/uhaf215 (PMC12582035; doi:10.1093/hr/uhaf215)

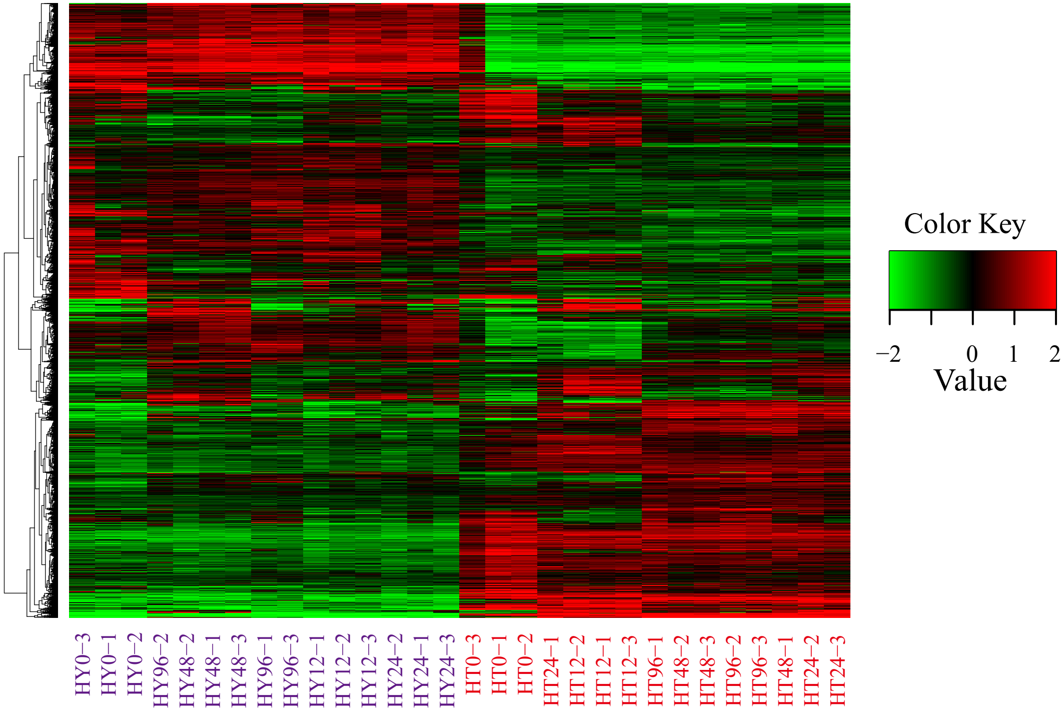

Color Key
−2
0
1
2
Value
HY0−3
HY0−1
HY0−2
HY96−2
HY48−2
HY48−1
HY48−3
HY96−1
HY96−3
HY12−1
HY12−2
HY12−3
HY24−2
HY24−1
HY24−3
HT0−3
HT0−1
HT0−2
HT24−1
HT12−2
HT12−1
HT12−3
HT96−1
HT48−2
HT48−3
HT96−2
HT96−3
HT48−1
HT24−2
HT24−3

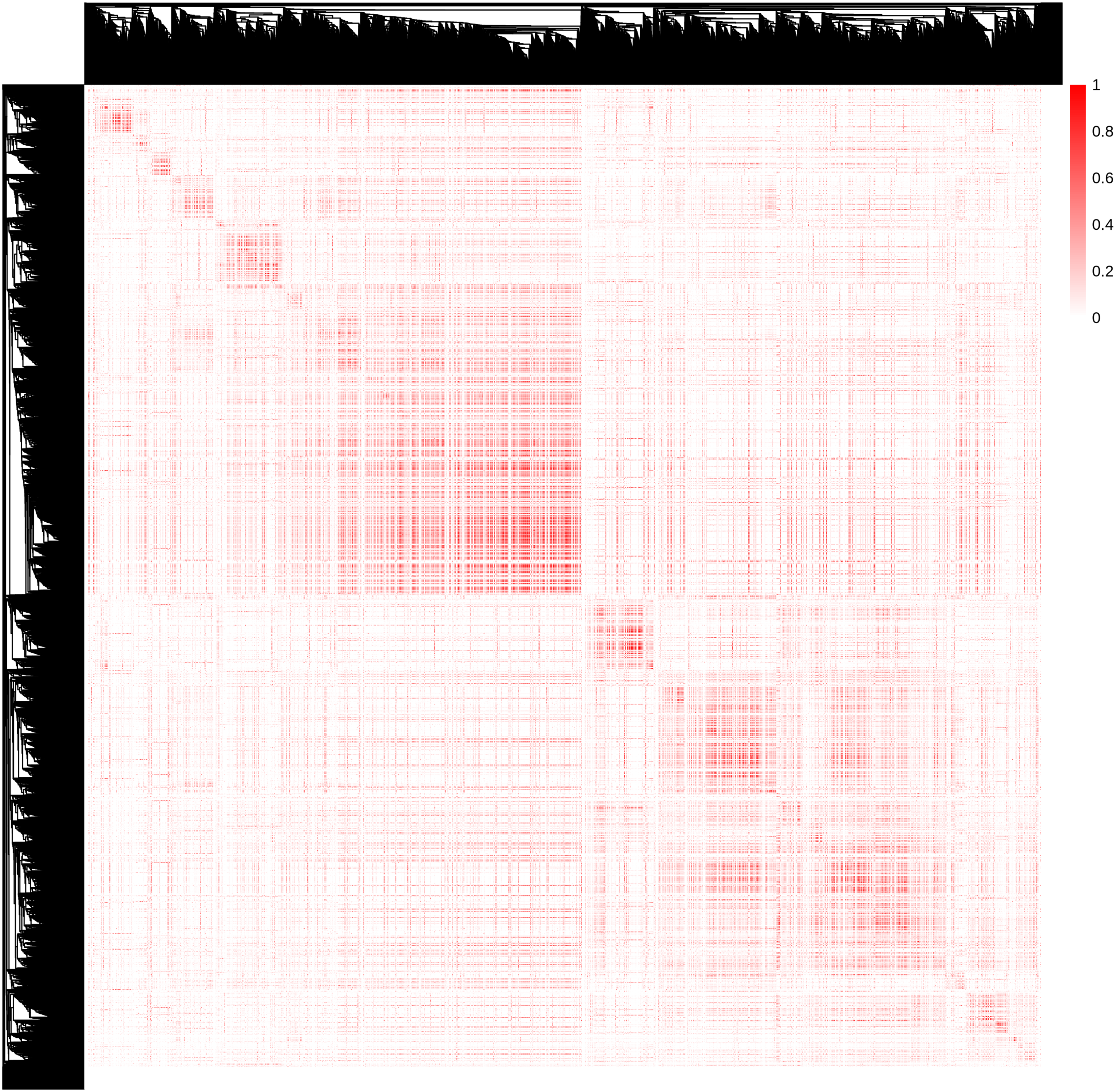

1
0.8
0.6
0.4
0.2
0

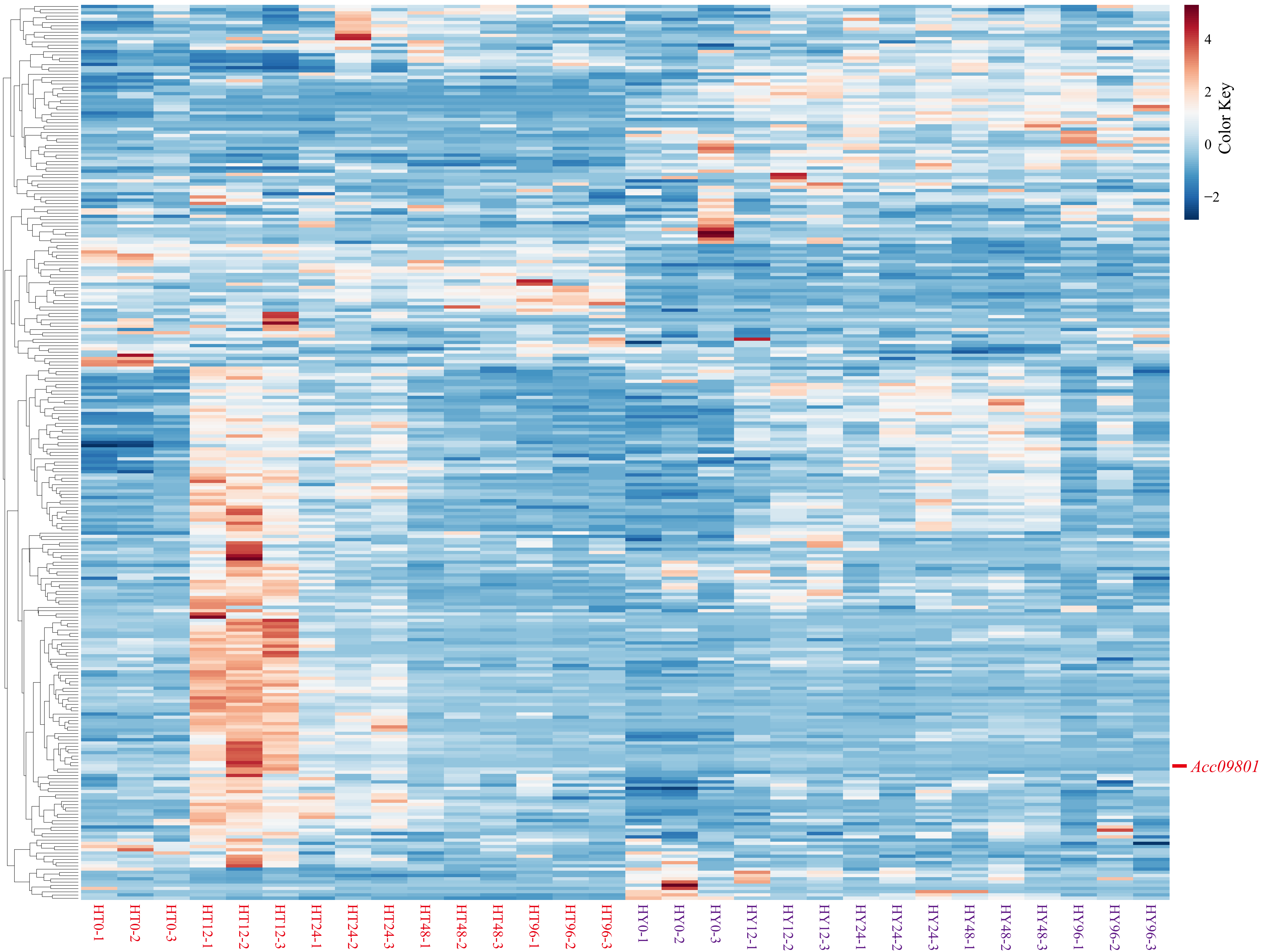

Color Key
4
2
0
−2
Acc09801
HT0-1
HT0-2
HT0-3
HT12-1
HT12-2
HT12-3
HT24-1
HT24-2
HT24-3
HT48-1
HT48-2
HT48-3
HT96-1
HT96-2
HT96-3
HY0-1
HY0-2
HY0-3
HY12-1
HY12-2
HY12-3
HY24-1
HY24-2
HY24-3
HY48-1
HY48-2
HY48-3
HY96-1
HY96-2
HY96-3

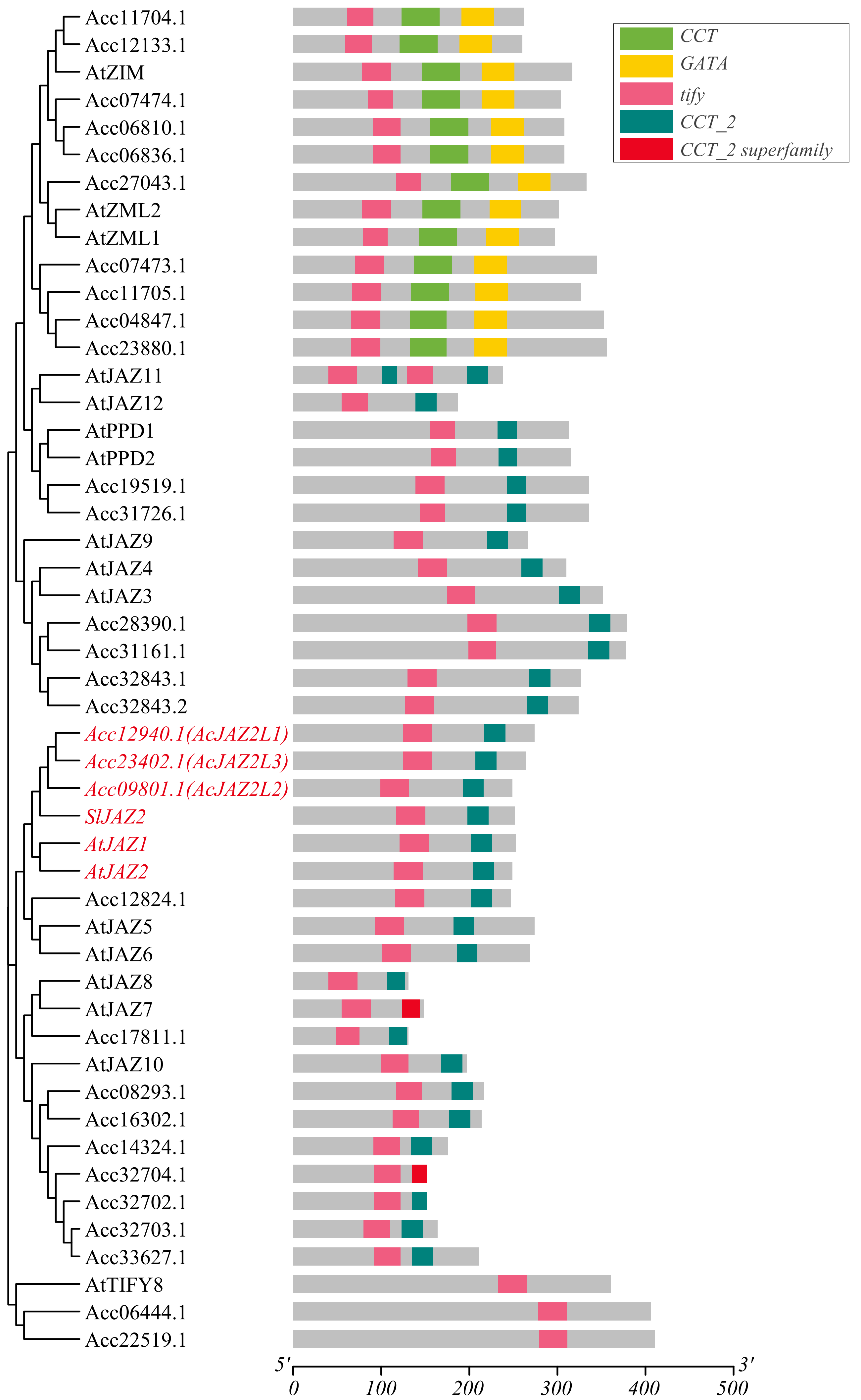
Acc11704.1
Acc12133.1
AtZIM
Acc07474.1
Acc06810.1
Acc06836.1
Acc27043.1
AtZML2
AtZML1
Acc07473.1
Acc11705.1
Acc04847.1
Acc23880.1
AtJAZ11
AtJAZ12
AtPPD1
AtPPD2
Acc19519.1
Acc31726.1
AtJAZ9
AtJAZ4
AtJAZ3
Acc28390.1
Acc31161.1
Acc32843.1
Acc32843.2
Acc12940.1(AcJAZ2L1)
Acc23402.1(AcJAZ2L3)
Acc09801.1(AcJAZ2L2)
SlJAZ2
AtJAZ1
AtJAZ2
Acc12824.1
AtJAZ5
AtJAZ6
AtJAZ8
AtJAZ7
Acc17811.1
AtJAZ10
Acc08293.1
Acc16302.1
Acc14324.1
Acc32704.1
Acc32702.1
Acc32703.1
Acc33627.1
AtTIFY8
Acc06444.1
Acc22519.1
CCT
GATA
tify
CCT_2
CCT_2 superfamily
5′
3′
0
100
200
300
400
500

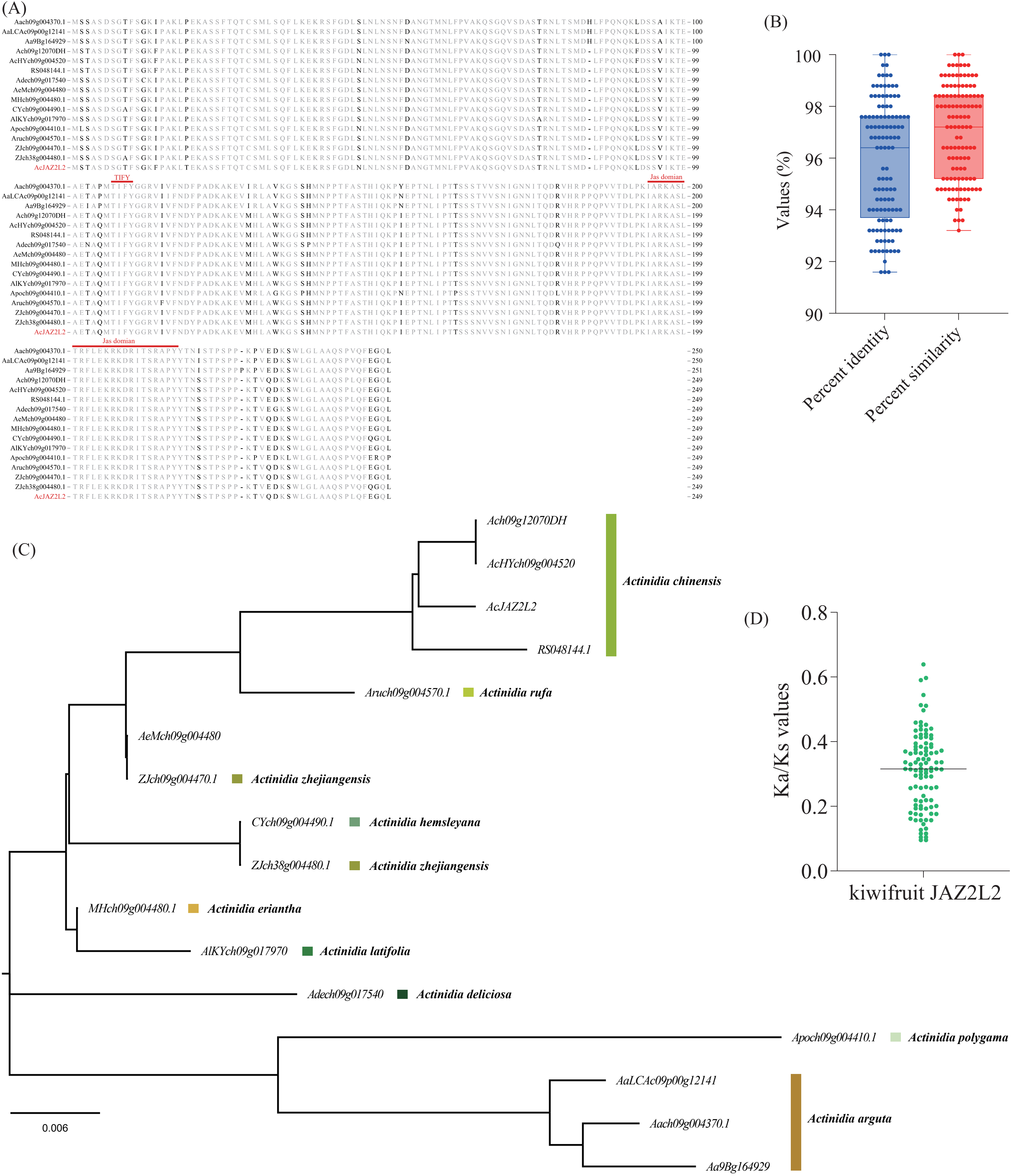

(A)
TIFY
Jas domian
Jas domian
Aach09g004370.1
AaLCAc09p00g12141
Aa9Bg164929
Ach09g12070DH
AcHYch09g004520
RS048144.1
Adech09g017540
AeMch09g004480
MHch09g004480.1
CYch09g004490.1
AlKYch09g017970
Apoch09g004410.1
Aruch09g004570.1
ZJch09g004470.1
ZJch38g004480.1
AcJAZ2L2
(B)
Values (%)
Percent identity
Percent similarity
(C)
Actinidia chinensis
Actinidia rufa
Actinidia zhejiangensis
Actinidia hemsleyana
Actinidia zhejiangensis
Actinidia eriantha
Actinidia latifolia
Actinidia deliciosa
Actinidia polygama
Actinidia arguta
0.006
(D)
Ka/Ks values
kiwifruit JAZ2L2

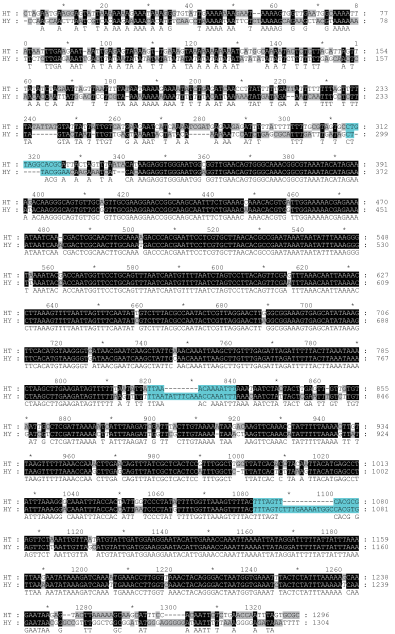

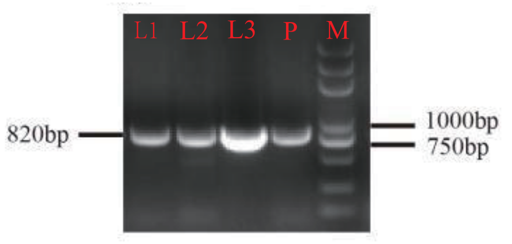
L1
L2
L3
P
M
820bp
1000bp
750bp

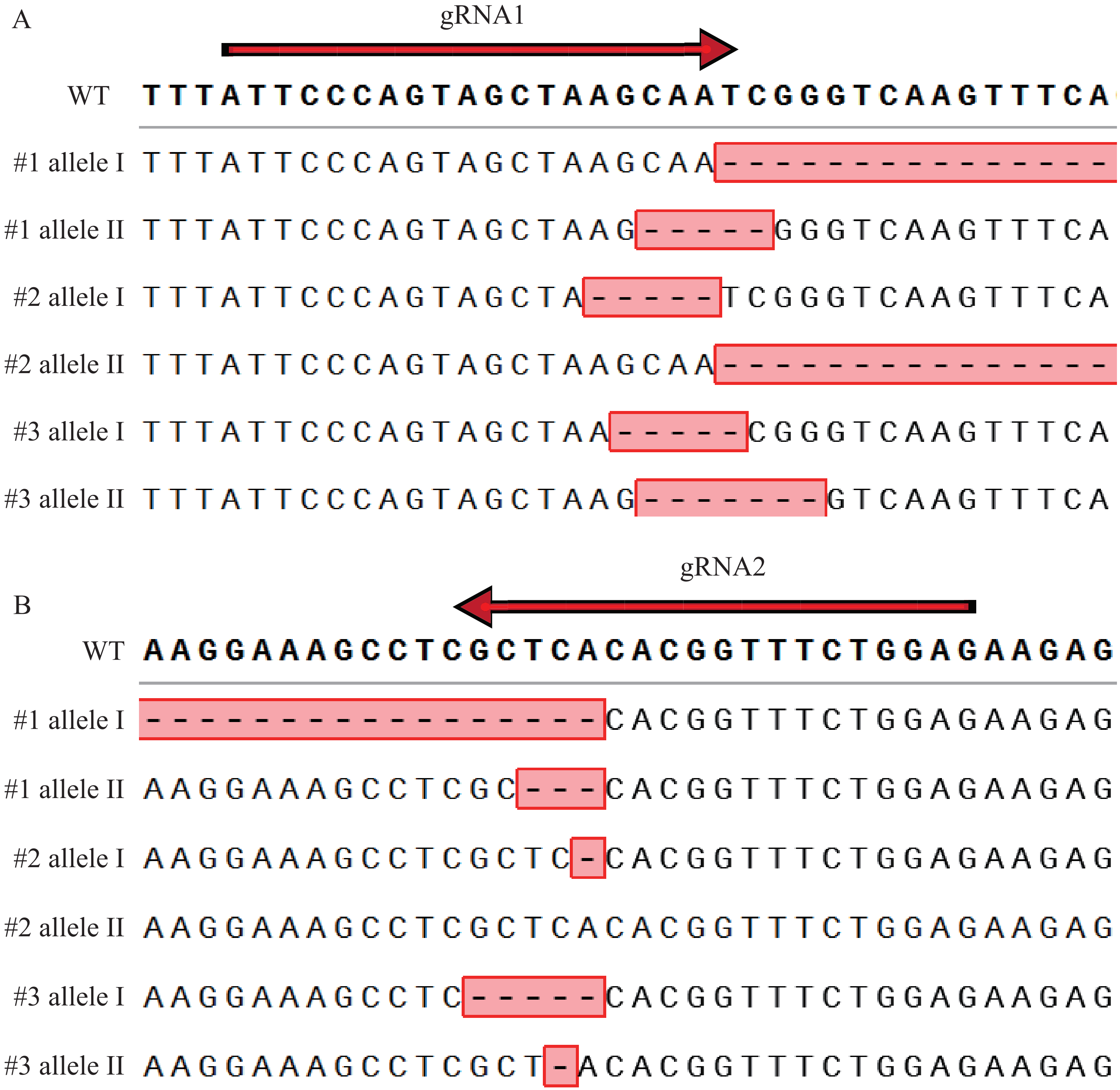

Figure S3. Identification of mutant alleles generated by PTG/Cas9-mediated editing at the gRNA1 and gRNA2 target sites in kiwifruit. The figure shows the wild-type (WT) allele and the two alleles (allele I and allele II) identified in the three mutant lines (#1, #2, #3). (A) Mutation profiles at the gRNA1 target site. (B) Mutation profiles at the gRNA2 target site.

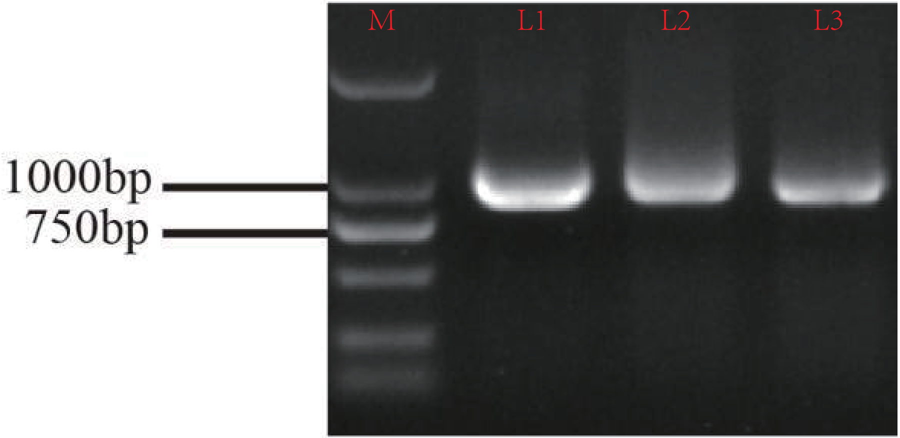
M
L1
L2
L3
1000bp
750bp

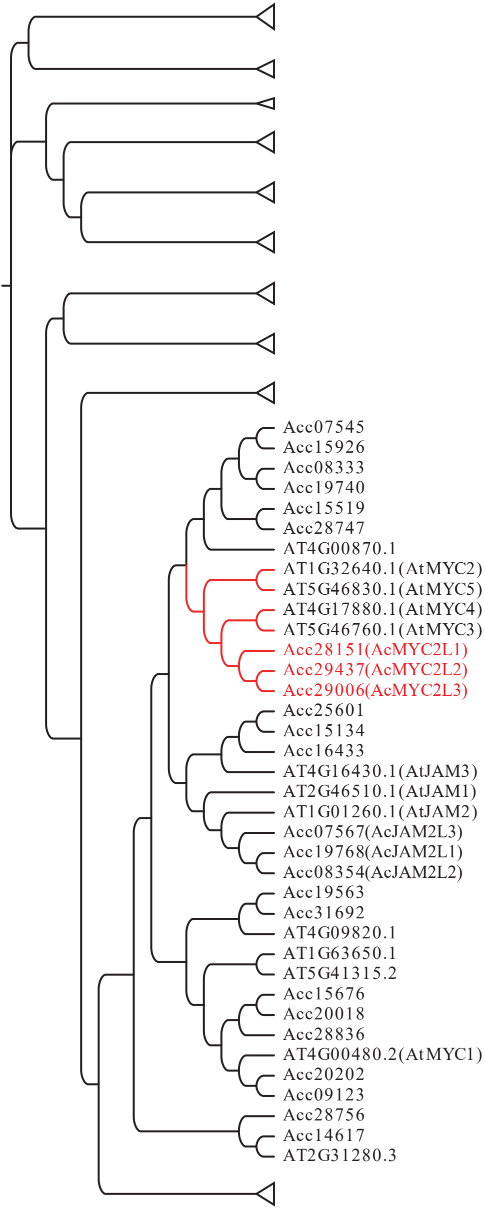

Acc07545
Acc15926
Acc08333
Acc19740
Acc15519
Acc28747
AT4G00870.1
AT1G32640.1(AtMYC2)
AT5G46830.1(AtMYC5)
AT4G17880.1(AtMYC4)
AT5G46760.1(AtMYC3)
Acc28151(AcMYC2L1)
Acc29437(AcMYC2L2)
Acc29006(AcMYC2L3)
Acc25601
Acc15134
Acc16433
AT4G16430.1(AtJAM3)
AT2G46510.1(AtJAM1)
AT1G01260.1(AtJAM2)
Acc07567(AcJAM2L3)
Acc19768(AcJAM2L1)
Acc08354(AcJAM2L2)
Acc19563
Acc31692
AT4G09820.1
AT1G63650.1
AT5G41315.2
Acc15676
Acc20018
Acc28836
AT4G00480.2(AtMYC1)
Acc20202
Acc09123
Acc28756
Acc14617
AT2G31280.3

A

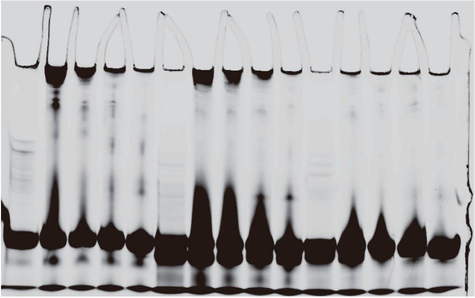

B

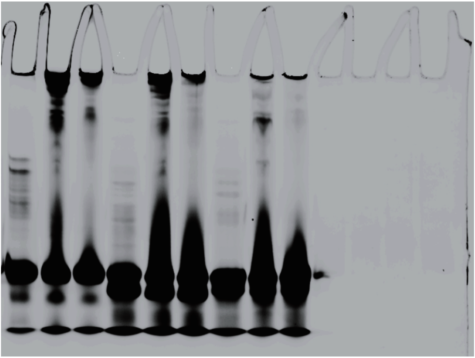

Supplement: Web_Material_uhaf215 [file web_material_uhaf215.zip › Supplemental Figures.pdf]
